# Intracellular deposits of amyloid-beta influence the ability of human iPSC-derived astrocytes to support neuronal function

**DOI:** 10.1186/s12974-022-02687-5

**Published:** 2023-01-03

**Authors:** Evangelos Konstantinidis, Benjamin Portal, Tobias Mothes, Chiara Beretta, Maria Lindskog, Anna Erlandsson

**Affiliations:** 1 Department of Public Health and Caring Sciences, Molecular Geriatrics, Uppsala University, 751 85 Uppsala, Sweden; 2 Department of Medical Cell Biology, Uppsala University, 751 23 Uppsala, Sweden

**Keywords:** Alzheimer’s disease, Amyloid-beta, Astrocytes, Electrophysiology, EPSCs, iPSCs, Neurons

## Abstract

**Background:**

Astrocytes are crucial for maintaining brain homeostasis and synaptic function, but are also tightly connected to the pathogenesis of Alzheimer’s disease (AD). Our previous data demonstrate that astrocytes ingest large amounts of aggregated amyloid-beta (Aβ), but then store, rather than degrade the ingested material, which leads to severe cellular stress. However, the involvement of pathological astrocytes in AD-related synaptic dysfunction remains to be elucidated.

**Methods:**

In this study, we aimed to investigate how intracellular deposits of Aβ in astrocytes affect their interplay with neurons, focusing on neuronal function and viability. For this purpose, human induced pluripotent stem cell (hiPSC)-derived astrocytes were exposed to sonicated Αβ_42_ fibrils. The direct and indirect effects of the Αβ-exposed astrocytes on hiPSC-derived neurons were analyzed by performing astrocyte–neuron co-cultures as well as additions of conditioned media or extracellular vesicles to pure neuronal cultures.

**Results:**

Electrophysiological recordings revealed significantly decreased frequency of excitatory post-synaptic currents in neurons co-cultured with Aβ-exposed astrocytes, while conditioned media from Aβ-exposed astrocytes had the opposite effect and resulted in hyperactivation of the synapses. Clearly, factors secreted from control, but not from Aβ-exposed astrocytes, benefited the wellbeing of neuronal cultures. Moreover, reactive astrocytes with Aβ deposits led to an elevated clearance of dead cells in the co-cultures.

**Conclusions:**

Taken together, our results demonstrate that inclusions of aggregated Aβ affect the reactive state of the astrocytes, as well as their ability to support neuronal function.

**Supplementary Information:**

The online version contains supplementary material available at 10.1186/s12974-022-02687-5.

## Background

Alzheimer’s disease (AD) is characterized by the deposition of plaques, mainly consisting of aggregated amyloid-β (Aβ), followed by the formation of neurofibrillary tangles [1]. These pathological features have been associated with the widespread neurodegeneration at later stages of AD. However, the exact cellular and molecular mechanisms by which Aβ induces neuronal dysfunction remain unclear. Although insufficient neuronal capacity is the main reason behind cognitive decline, other cell types in the AD brain, including glial cells, are also severely affected [2, 3]. Growing evidence indicates that incomplete degradation of Aβ aggregates by glial cells, and chronic inflammation could be driving forces of AD pathology and participate in disease progression [4, 5]. Astrocytes are abundant in the human brain and are crucial for maintaining brain homeostasis by providing physical, energetic, metabolic and trophic support to neurons and other brain cell types [6]. Hence, functionally impaired astrocytes may be devastating for the brain health in general and neuronal activity in particular.

The term tripartite synapse was first proposed in the 1990s to highlight the importance of astrocytes in the function of neuronal networks [7]. Since then, this bidirectional communication between neurons and astrocytes has been further explored and characterized [8, 9]. The release of neurotransmitters within the synaptic cleft elicits an astrocytic calcium-mediated response, which in turn leads to secretion of trophic factors associated with neuronal maturation and synapse formation [10, 11], as well as neuroactive molecules known as gliotransmitters [12]. These molecules that include glutamate, D-serine, ATP and others, play an important role in regulating synaptic transmission and neuronal activity [13–15].

Reactive astrocytes effectively engulf dead cells, damaged synapses and protein aggregates [16–18]. Importantly, our previous studies demonstrate that astrocytes store, rather than degrade ingested Aβ aggregates [19–21]. A high proportion of the accumulated Aβ is N-terminally truncated, indicating that it is processed and modified by the astrocytes, although not completely digested. This incomplete degradation causes severe cellular stress and may result in spreading of pathological Aβ aggregates. We and others have demonstrated that stressed astrocytes transfer protein aggregates to neighboring cells via thin protrusions called tunneling nanotubes (TNTs) [21–24]. Moreover, our previous data show that incomplete degradation of Aβ in murine astrocytes results in the secretion of truncated, potentially toxic Aβ peptides [20, 25]. Recent reports suggest that neuronal dysfunction and cognitive decline in AD are not due to low synaptic activity as formerly believed, but rather due to hyperactivity at the early stages of the disease [26, 27]. However, the role of astrocytes in the AD-related neuronal hyperactivity remains to be elucidated.

Over the years, mice have been the most common source of cultured astrocytes and neurons, and have facilitated the deciphering of cellular functions and involvement in disease progression. However, mouse cells differ in many aspects from human cells in terms of morphology, gene expression and response to disease-associated stimuli [28]. Thus, the development of human induced pluripotent stem cell (hiPSC)-derived in vitro models for neurodegenerative diseases, including AD, has expanded the ability to study complex pathways and understand disease-specific pathological mechanisms [29]. In this study, we have utilized previously established protocols for astrocytic and neuronal differentiation, with some modifications, in order to obtain mature cells to recapitulate aspects of Aβ-associated AD pathology in a human model system [30, 31]. Our overall aim was to investigate how inclusions of aggregated Aβ in astrocytes affect their homeostatic function and consequently influence nearby neurons in terms of cellular viability and synaptic activity.

## Materials and methods

### Culture of human iPSC-derived astrocytes and neurons

Human astrocytes were derived from long-term neuroepithelial-like stem (ltNES) cells, generated from human induced pluripotent stem cells (Cntrl9 II cell line) as previously described, with some modifications [30, 31]. Briefly, ltNES cells were plated at 60,000 cells/cm^2^ on 100 μg/ml poly-L-ornithine hydrobromide (#P3655, Merck, Darmstadt, Germany) and 4 μg/ml Laminin2020 (#L2020, Merck) double-coated culture vessels in astrocyte differentiation medium; Advanced DMEM/F12 (#11540446) supplemented with 1% penicillin–streptomycin (#11548876), 2% B27 supplement (#11530536), 1% non-essential amino acids (#11140050), and 1% L-glutamine (#25030-024) (all from Thermo Fisher Scientific, MA, USA). The following factors were added fresh to the medium before use: recombinant human fibroblast growth factor-basic (bFGF) 10 ng/ml (#11390832, Thermo Fisher Scientific), heregulin 10 ng/ml (#SRP3055, Merck), activin A 10 ng/ml (#120-14E, PeproTech, NJ, USA), insulin-like growth factor 1 (IGF-1) 200 ng/ml (#SRP3069, Merck) and human ciliary neurotropic factor (CNTF) 20 ng/ml (#PHC7015, Thermo Fisher Scientific). The medium was changed every other day and astrocytes were passaged using Trypsin–EDTA (#15400054, Thermo Fisher Scientific) once cells reached 80% confluency. For experiments, the astrocytes were seeded at a density of 2000 (co-cultures) or 5000 (monocultures) cells/cm^2^, resulting in a 20% and 50% confluency, respectively. Cells from 30 days in vitro (DIV) to 66 DIV were used in the study.

Neuronal differentiation was performed as previously described, with some modifications [30, 32]. Briefly, ltNES cells were plated at 20,000 cells/cm^2^ on 100 μg/ml poly-L-ornithine hydrobromide and 20 μg/ml Laminin2020 double-coated culture vessels in neuronal differentiation medium #1; DMEM/F12 + Glutamax (#11540446, Thermo Fisher Scientific) supplemented with 1% penicillin–streptomycin, 1% B27 supplement and 1% N2 supplement (#11520536, Thermo Fisher Scientific). After day 9, neuronal differentiation medium was combined with Neurobasal (#11570556, Thermo Fisher Scientific) supplemented with 1% Glutamax, 1% penicillin–streptomycin and 1% B27 supplement at a 1:1 ratio (neuronal differentiation medium #2). Cells from 30 to 66 DIV were used in the study.

### *Production of Aβ*_*42*_* fibrils (Aβ-F)*

Fluorescent HiLyte™ Fluor 555 labeled human Aβ_42_ monomers (Aβ_42_-555; #60480-01, AnaSpec, CA, USA) were aggregated into insoluble fibrils according to a well-established protocol. Briefly, the synthetic Aβ_42_ monomers were dissolved in a 10 mM NaOH and 10× PBS (#11530486, Thermo Fisher Scientific) solution to a final concentration of 2 mg/ml. The Aβ_42_ samples were incubated on a shaker at 1500 rpm, 37 °C for 4 days. Finally, the resulting Αβ-F were diluted in sterile 1× PBS to the final concentration of 0.5 mg/ml and sonicated at 20% amplitude, 1 s on/off pulses for 1 min (#VCX130, Vibra Cell sonicator, Sonics, CT, USA).

### Exposure of astrocytes to Aβ-F

Astrocytes were exposed to 200 nM of sonicated Aβ-F in astrocyte differentiation medium for 2 days. The reason for using sonicated Aβ-F was to enable cellular uptake. Control cultures received medium without Aβ-F. At day 3 after Aβ exposure, the cells were washed and then cultured in Aβ-free medium for 6 (2d + 6d), 12 (2d + 12d) or 33 (2d + 33d) days, for transfer to co-cultures, isolation of extracellular vesicles (EVs) or conditioned media experiments, respectively.

### Co-culture of human iPSC-derived neurons and astrocytes

Neurons were cultured for 30 days as described above, before control astrocytes or astrocytes that had been exposed to Aβ-F (2000 cells/cm^2^) were added to the culture (1:10 astrocyte to neuron ratio). Neuronal differentiation medium #2 was used for co-culture maintenance. The co-cultures were analyzed 7–33 days after addition of the astrocytes, depending on the experimental setup.

### Conditioned media experiments

For conditioned media experiments, astrocytes were seeded at a density of 5000 cells/cm^2^ (~ 5.6 × 10^5^ cells) in 5 ml of astrocyte differentiation medium, on a 10-cm culture dish. Control astrocytes and astrocytes exposed to Aβ-F for 2 days, were washed and kept in culture for additional 12 days. Every 3 days, all the conditioned medium from treated and control astrocytes was collected and diluted 1:1 with neuronal differentiation medium #2 before addition to neuronal monocultures. Thereafter, the cells were used for electrophysiological recordings or fixed for immunocytochemistry.

### Isolation of extracellular vesicles

For isolation of EVs, approximately 1 × 10^6^ astrocytes were seeded in 5 ml of astrocyte differentiation medium, on a 10-cm culture dish. Control astrocytes and astrocytes exposed to Aβ-F for 2 days, were washed and incubated for additional 6 or 12 days without Aβ. The conditioned media were collected at 6 days and 12 days following the Aβ exposure (2d + 6d and 2d + 12d). The media from the two timepoints were pooled prior to ultracentrifugation. The pooled medium samples for each treatment and cell culture batch were centrifuged at 300 ×*g* for 5 min to remove any free-floating cells, followed by another centrifugation at 2000 ×*g* for 10 min to remove any remaining cell debris. The supernatants were collected and transferred to ultracentrifuge tubes and centrifuged at 135,000 ×*g* at 4 °C for 90 min to isolate EVs, including larger microvesicles and exosomes. The vesicle pellets were resuspended in either neuronal medium and added to neurons or 1× PBS for TEM.

### Extracellular vesicle experiments

For cell exposure experiments, EVs were isolated from control and Aβ-F-treated astrocytes as described above. The vesicle pellets were resuspended in 100 μl neuronal differentiation medium #2 and added to neuronal monocultures at day 30, which were cultured for 5 additional days before fixation and further analyses.

### Immunocytochemistry

Cells were fixed with 4% paraformaldehyde (PFA) (#P6148, Merck) in 1× PBS for 15 min at RT and washed 3 times with 1× PBS. Prior to antibody incubation, the cells were permeabilized and blocked with 0.1% Triton X-100 or saponin in 1× PBS with 5% normal goat serum (NGS) (#S-1000, Vector Laboratories, CA, USA) for 30 min at RT. Primary antibodies (Table 1) were diluted in 0.1% Triton X-100 or saponin in 1× PBS with 0.5% NGS and the coverslips were incubated with the antibody solution for 1–2 h at RT. The coverslips were washed 3 times with 1× PBS and then incubated with secondary antibodies (Table 1) diluted in 0.1% Triton X-100 or saponin in 1× PBS with 0.5% NGS for 45 min at 37 °C. The coverslips were washed 3 times with 1× PBS and mounted on microscope slides using EverBrite hardset medium with DAPI (#230032, Biotium, CA, USA) and/or Vectashield hardset antifade mounting medium with phalloidin (#H-1600-10, Vector Laboratories). Images were captured using the fluorescence microscope Zeiss Axio Observer Z1 (ZEISS microscopy, Jena, Germany).

**Table 1:** Antibodies used in this study

Manufacturer	Target	Antibody	Dilution
Merck	Vimentin	AB5733	1:200
Merck	S100β	S2532	1:200
Abcam	GFAP	ab4674	1:400
Abcam	Synaptophysin	ab52636	1:200
Merck	MAP2	AB5622	1:200
Novus Biologicals	GLAST/EAAT1	NB100-1869	1:400
Thermo Fisher Scientific	Goat anti-Ms	AlexaFluor 488, 555	1:200
Thermo Fisher Scientific	Goat anti-Rb	AlexaFluor 488, 647	1:200
Thermo Fisher Scientific	Goat anti-Ch	AlexaFluor 555, 647	1:200

### Cell viability assays and quantification of dead/live cells

TUNEL staining was used to visualize dead cells in the fixed coverslips, according to the manufacturer’s instructions (#11767891910, Merck). Briefly, fixed cells were incubated with TUNEL Label mixed with TUNEL Enzyme for 1 h at 37 °C. Quantification of dead/live cells was performed using a custom pipeline in CellProfiler™ (www.cellprofiler.org). The pipeline split the DAPI (blue) and TUNEL (green) channels, identified the live and dead nuclear staining via specific size and circularity parameters and then overlayed the images in order to calculate the percentage of apoptotic (green) cells.

### Transmission electron microscopy

Transmission electron microscopy was performed as previously described [20].

Sonicated Aβ-F before and after sonication were diluted 1:10 in 1× PBS and dropped onto carbon coated 300-mesh copper grids, negatively stained with 1% uranyl acetate for 5 min and air dried. The samples were imaged using a Tecnai G2 transmission electron microscope (TEM, FEI company).

Extracellular vesicle samples were mixed with an equal volume of 4% paraformaldehyde, added onto a formvar and carbon coated 200-mesh grid (Oxford 11 Instruments) and incubated for 20 min. After incubation, the grid was dried and washed three times with 1× PBS, followed by eight washes in Milli-Q water. The samples were stained in a drop of uranyl-oxalate (pH 7), for 5 min, after which they were stained with a drop of uranyl acetate 4% (pH 4) with 2% methylcellulose on ice for 10 min. The dried grids were imaged using a Tecnai G2 transmission electron microscope (TEM, FEI company) with an ORIUS SC200 CCD camera and Gatan Digital Micrograph software (both from Gatan Inc., CA, USA).

### Patch-clamp experiments

Cells grown on glass coverslips were placed in a patch-clamp chamber (#RC-27, Warner Instruments, CT, USA) and perfused with an extracellular solution containing (in mM) 110 NaCl, 1 NaH_2_PO_4_, 4 KCl, 25 4-(2-hydroxyethyl) piperazine-1-ethanesulfonic acid (HEPES), 100 glucose, 1.2 MgCl_2_ and 1.5 CaCl_2_ (pH 7.4) at a rate of 2–3 ml per min and a temperature of 32 °C. Borosilicate glass pipettes with a tip resistance of 3–7 MOhm were used for patching the cells. The glass pipettes were filled with a solution containing (in mM): 110 K-gluconate, 10 KCl, 4 Mg-ATP, 10 Na_2_-phosphocreatine, 0.3 Na-GTP, 10 HEPES and 0.2 ethylene glycol tetra acetic acid (EGTA) (pH 7.2–7.4; 270–290 mOsm). Resting membrane potential was measured immediately after opening the patched cell. Excitatory post-synaptic currents (EPSCs) were recorded either in the absence (sEPSCs) or presence (mEPSCs) of 0.5 μM of tetrodotoxin (TTx) in the extracellular solution to block action potentials. Access resistance was monitored throughout the recordings, only data displaying < 30% variation were included. Data was acquired using an Ag/AgCl electrode and a Multiclamp 700B amplifier digitized with Digidata 1440A (Molecular Devices CA, USA). Recordings were monitored using the Clampex software 10.0 (Molecular Devices).

### Analysis of excitatory post-synaptic potentials (EPSCs)

Traces were analyzed with the Easy Electrophysiology software (v2.3.3-beta; https://www.easyelectrophysiology.com). We first used probe recordings (three recordings of one minute each) from primary neuronal cultures to generate and refine a template from representative events corresponding to an average of 10 to 20 detected EPSCs. Event detection in hiPSC-derived neuronal cultures was then run on a semi-automatic method as follows: each event detected was fitted to the previously generated template. When the amplitude of a detected event was the same as the template one, the event was saved for further analysis. A minimum amplitude threshold of 10 pA was applied. All events that were detected between 0 and 10 pA were discarded. Each recording was analyzed in a period of 1 min.

### Statistical analyses

Statistical analyses were conducted in GraphPad Prism v9.3.1 and IBM^®^ SPSS^®^ Statistics (v28). A summary of all statistical analyses is available in Additional file 1: Table S1. For every measured variable, we performed a Shapiro–Wilk and a Brown–Forsythe test to assess normal data distribution and the variance of standard deviation in all the groups to determine the appropriate type of statistical test. The level of significance for all the graphs is: ns = non-significant, *p < 0.05, **p < 0.01, ***p < 0.001 and ****p < 0.0001. Results are presented as mean ± S.E.M.

### Viability assays and immunocytochemistry quantification

A minimum of 20 images (each consisting of a 5 × 5 tiled image) per group for each experimental condition was captured at 20× magnification from a total of three independent experiments. The intra-experiment variability is larger or equal to the inter-experiment variability and therefore the replicates are not considered as independent data. The IntDen fluorescence intensity signal was measured using the ImageJ software.

### Electrophysiology experiments

A very high variation was observed within groups, especially regarding the frequency of both sEPSCs and mEPSCs, thus the R package “cvequality” (v0.1.4) was used to analyze potential differences in the coefficient of variation (Additional file 1: Table S2) [33]. An asymptotic test for equality of the coefficient of variation revealed no differences across groups for the frequency of sEPSCs (*χ*^2^ = 0.620, *p* = 0.959) [34]. However, the coefficient of variation in the amplitude of sEPSCs was statistically different across groups (*χ*^2^ = 12.44, *p* = 0.014). The same analysis revealed no differences in the coefficient of variation neither in the frequency nor amplitude of mEPSCs (*χ*^2^ = 2.15, *p* = 0.706; *χ*^2^ = 5.74, *p* = 0.218, for frequency and amplitude of mEPSCs, respectively). Overall, the variation was similar between the groups, regardless of the considered variable. While big variations usually mask a potential statistical effect, this was not the case in our study, pointing out the robustness of the analysis.

## Results

### A cell-culture model of AD based on human iPSC-derived neurons and astrocytes

**Fig. 1:**
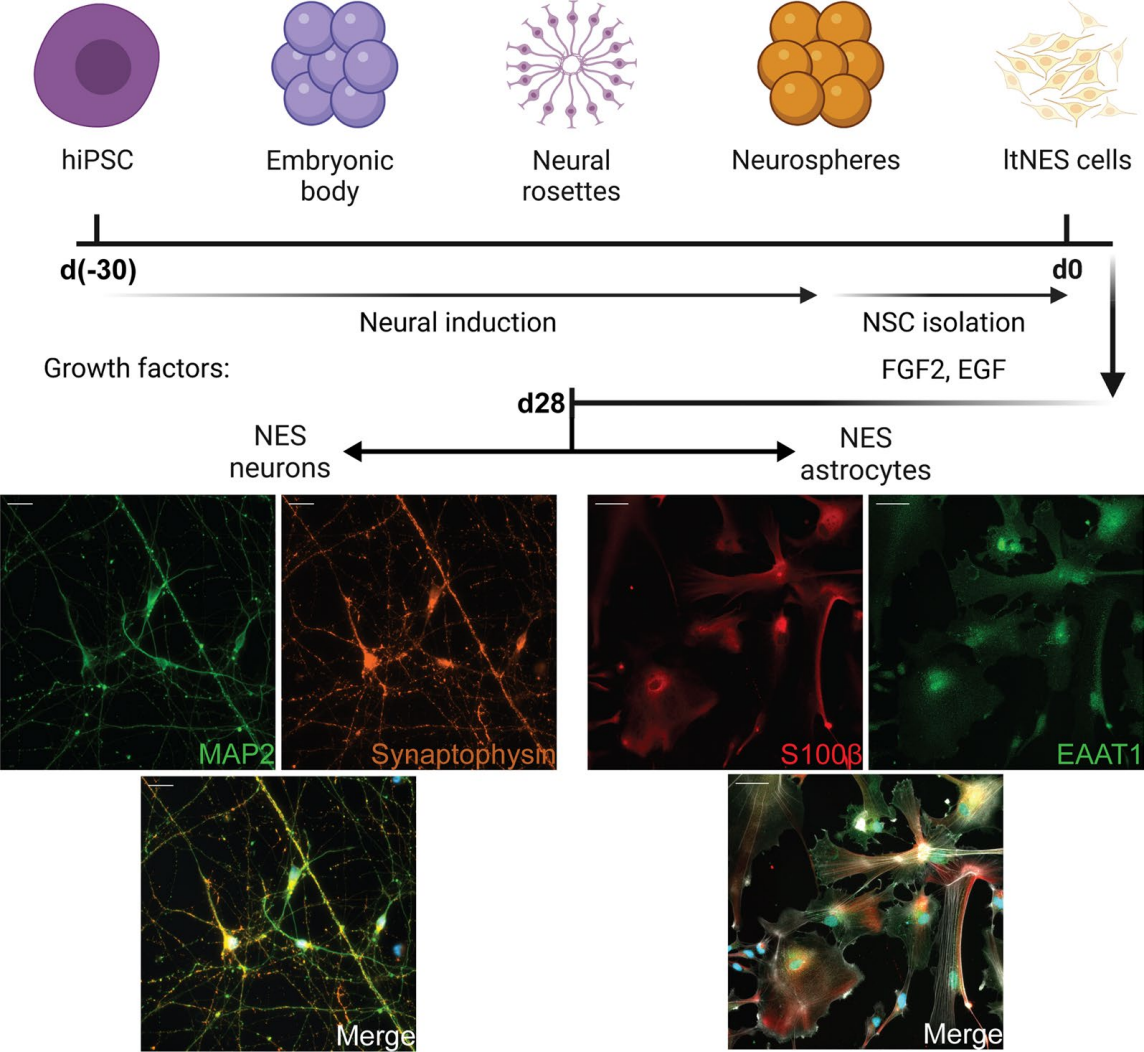
Differentiation timeline for hiPSC-derived neurons and astrocytes. Both differentiation pathways began from the hiPSC stage and followed the same neural induction process until the ltNES stage. Neuronal induction was accomplished in 28 days using neuronal differentiation media without extra growth factors. At day 28, the neurons expressed MAP2 (green) and synaptophysin (orange) (Scale bar: 20 μm). Astrocyte induction required specific factors that promote differentiation of ltNES cells to functional, mature astrocytes. At day 28, the astrocytes expressed S100β (red) and EAAT1 (green), while phalloidin (white) was used to stain the cytoskeleton (scale bar: 50 μm)

In order to investigate how Aβ pathology affects the crosstalk between human neurons and astrocytes, we designed an experimental setup based on hiPSC-derived cell cultures. Human neurons and astrocytes were obtained from the same hiPSC line, using previously established differentiation protocols with some modifications [30, 31]. After 28 days of differentiation, the neurons expressed the specific markers MAP2 and synaptophysin, while the astrocytes expressed S100β and EAAT1 (Fig. 1A, B).

**Fig. 2:**
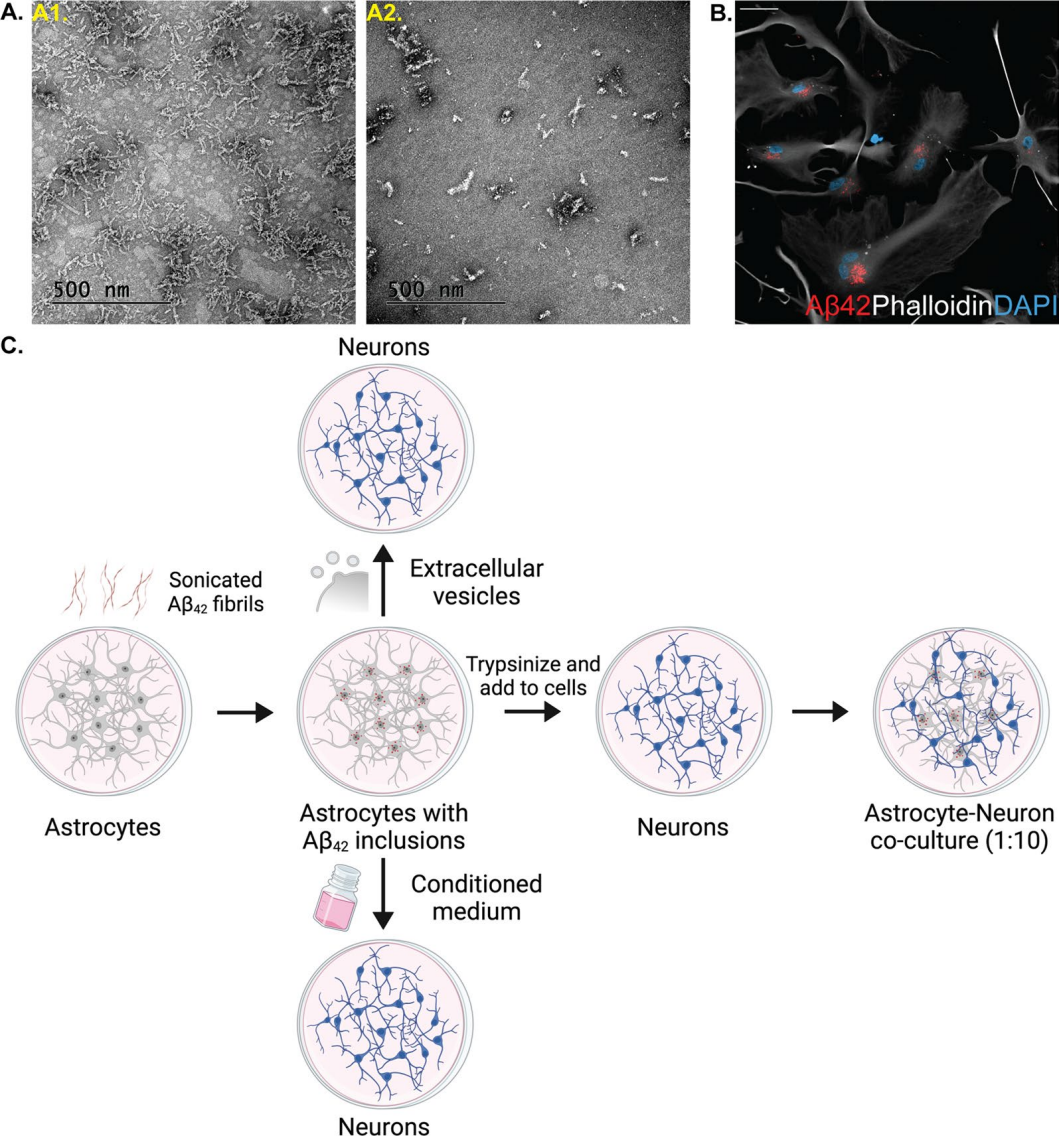
Human astrocytes ingest and accumulate sonicated Aβ-F. **A** Representative transmission electron microscopy images of Aβ-F before (A1) and after (A2) sonication (scale bar: 500 nm). **B** Fluorescent images of Aβ-F-exposed astrocytes. The Aβ-F (red) deposits were present in astrocytes (white) throughout the experiment (scale bar: 20 μm). **C** Experimental setup of the study (created with www.Biorender.com)

We know from our previous work that murine and human astrocytes effectively engulf exogenously added Aβ aggregates, but store, rather than degrade the ingested material [20, 21]. Here, we induced astrocytic Aβ pathology following the same experimental paradigm, by exposing fully differentiated hiPSC-derived astrocytes to sonicated Aβ-F (Fig. 2A). After 48 h we could not detect any remaining free-floating Αβ-F in the medium, as all sonicated fibrils had been ingested and accumulated by the astrocytes (Fig. 2B).

To model AD pathogenesis and explore if the intracellular storage of Aβ-F in astrocytes affects their interplay with neurons, we designed experiments based on direct neuron-to-astrocyte contact as well as indirect interactions through additions of astrocytic conditioned media or extracellular vesicles to pure neuronal cultures (Fig. 2C).

### Increased clearance of dead cells in co-cultures of neurons and Aβ-exposed astrocytes

**Fig. 3:**
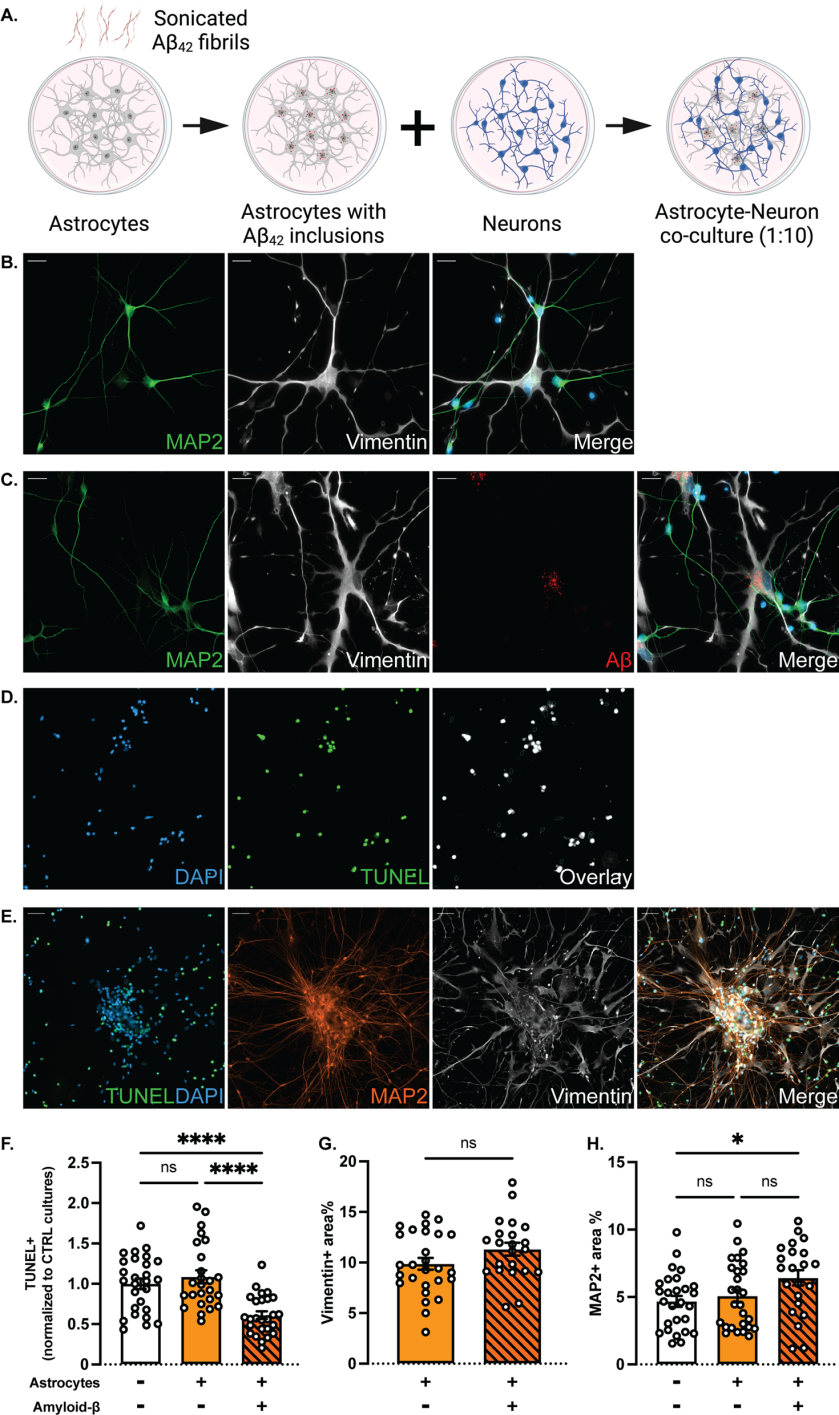
Co-cultures of neurons and Aβ-exposed astrocytes contain fewer apoptotic cells. **A** Study design to investigate the effects of cell-to-cell interaction between control or Aβ astrocytes and neurons (created with www.Biorender.com). **B**, **C** Representative images of control and Aβ co-cultures, respectively (scale bar: 20 μm). **D** Images generated by the apoptotic cell analysis pipeline in CellProfiler™. Live cells (blue) and dead cells (green) were overlayed to provide the dead/live percentage. **E** Astrocytes (white) surrounded the clumps of neuronal cell corpses (green) and live neurons (orange) (scale bar: 50 μm). **F** The percentage of apoptotic cells was decreased in Aβ co-cultures compared to control co-cultures and neuronal monocultures. **G** Astrocytic area% as measured by vimentin + staining displayed no difference between control and Aβ co-cultures. **H** Neuronal area% as measured by MAP2 + staining was increased in Aβ co-cultures, compared to control co-cultures and neuronal monocultures (Results in all graphs are presented as mean ± S.E.M. from three independent experiments; ns = non-significant, *p < 0.05, ****p < 0.0001)

Intracellular Aβ deposits are a strong stressor for the astrocytes and may influence their crosstalk with neurons in several ways [20]. To better understand how Aβ pathology affects the direct interplay between the two cell types, we generated co-culture systems consisting of astrocytes, with or without Aβ inclusions, and healthy neurons. The neurons and astrocytes were differentiated separately and, following Aβ exposure, the control or Aβ astrocytes were seeded onto the neuronal monocultures at a ratio of one astrocyte to ten neurons (1:10) (Fig. 3A). Importantly, the astrocytes maintained the Aβ inclusions following trypsinization and reseeding. After 7 to 10 days of co-culture, the morphology and integration of the astrocytes into the neuronal network were analyzed using immunocytochemistry. Both cell types remained mature and displayed lineage-specific cell markers (Fig. 3B, C). Interestingly, we could detect clear connections between the majority of the astrocytes and neurons in the culture, indicating pronounced direct communication (Fig. 3C).

To evaluate if the presence of control astrocytes or astrocytes with Aβ inclusions affected the overall neuronal viability, we performed TUNEL assays and developed a pipeline within the CellProfiler™ environment in order to analyze the data (Fig. 3D). During the 28-day neuronal differentiation, there is typically a high degree of cell death. In the absence of glial cells, cell corpses remained within the culture and formed noticeable clumps together with viable neurons. When added, astrocytes were evenly distributed across the cultures, albeit with an increased preference towards the areas with the clumps (Fig. 3E).

The TUNEL analysis revealed a clear decrease in the percentage of apoptotic cells in co-cultures with Aβ-containing astrocytes, compared to the control co-cultures and neuronal monocultures (Fig. 3F). The cell corpses were apparently more effectively cleared by the Αβ-exposed astrocytes than by the control astrocytes, suggesting that the presence of Αβ deposits made the astrocytes more phagocytic and more degradation prone. Furthermore, we analyzed the area percentage (area%) of each cell type in the different cultures by quantifying the vimentin and MAP2-positive staining for the astrocytes and neurons, respectively. We did not observe any difference in astrocytic area% between the control and Αβ co-cultures (Fig. 3G). However, we noted an increase in the MAP2-positive area% in the Aβ co-cultures, which is in line with the increased clearance of apoptotic neurons (Fig. 3H and Additional file 1: Fig. S1).

### Conditioned medium from control astrocytes, but not from Aβ-exposed astrocytes promotes viability of neuronal monocultures

**Fig. 4:**
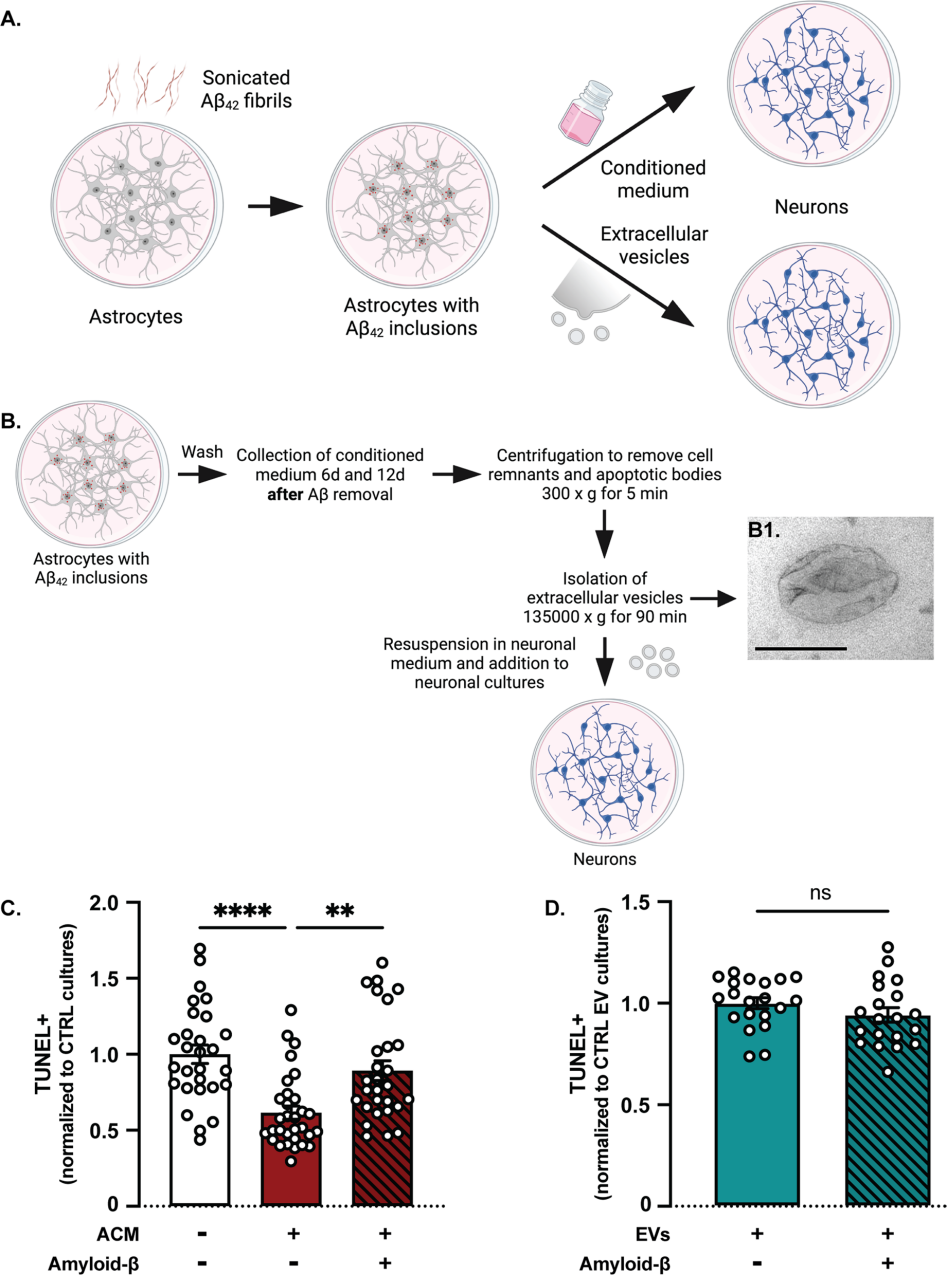
Conditioned media, but not EVs affect overall viability in neuronal monocultures. **A** Study design to investigate the effect of conditioned media and EVs from control and Aβ-exposed astrocytes on neuronal monocultures (created with www.Biorender.com). **B** EVs were isolated from astrocytic media via a series of centrifugation and ultracentrifugation steps. (B1) TEM analysis revealed characteristic EV morphology (scale bar: 200 nm). **C** Overall viability was increased in neuronal monocultures that received ACM from control astrocytes, compared to control cultures and cultures that received Aβ ACM. **D** No difference in viability was observed in neuronal monocultures treated with EVs from control or Aβ-exposed astrocytes [Results are presented as mean ± S.E.M. from three (**C**) and two (**D**) independent experiments, respectively; values are normalized to the control neuronal monocultures and control EV monocultures for **C** and **D**, respectively; ns = non-significant, **p < 0.01, ****p < 0.0001]

Next, we sought to investigate whether addition of astrocytic conditioned media (ACM) or EVs from control or Aβ-containing astrocytes would have a distinct effect on neuronal monocultures similar to the co-culture setup (Fig. 4A). To that end, Aβ-exposed astrocytes were thoroughly washed and cultured for additional 12 days in medium without Aβ. During the 12 days, conditioned media were collected from control and Aβ astrocytes every third day and added to neuronal monocultures. The EVs were isolated by sequential centrifugations of pooled astrocytic media collected at days 6 and 12 (Fig. 4B).

Following TUNEL labeling, we used the CellProfiler™ pipeline to calculate the percentage of apoptotic cells in neuronal monocultures treated with ACM or EVs from control and Aβ-exposed astrocytes. In contrast to the co-culture setup, we observed a significant increase in cell death in monocultures that received ACM from Aβ astrocytes compared to those that received ACM from control astrocytes (Fig. 4C). There was also a significant decrease in the percentage of apoptotic cells in monocultures that received control ACM, compared to monocultures that did not receive any conditioned media, suggesting a potential benefit in overall survival and wellbeing due to factors secreted by control astrocytes, but not Aβ astrocytes. Monocultures treated with EVs isolated from control or Aβ astrocytes, did not show any difference in viability, indicating that the trophic factors were not delivered through vesicles, but rather secreted directly into the medium (Fig. 4D). To further decipher the differences between the direct and indirect effects of astrocytic presence, we decided to explore the functional characteristics of the neuronal monocultures in terms of maturation and synaptic activity.

### Neuronal monocultures require 66 days to achieve functional maturation

**Fig. 5:**
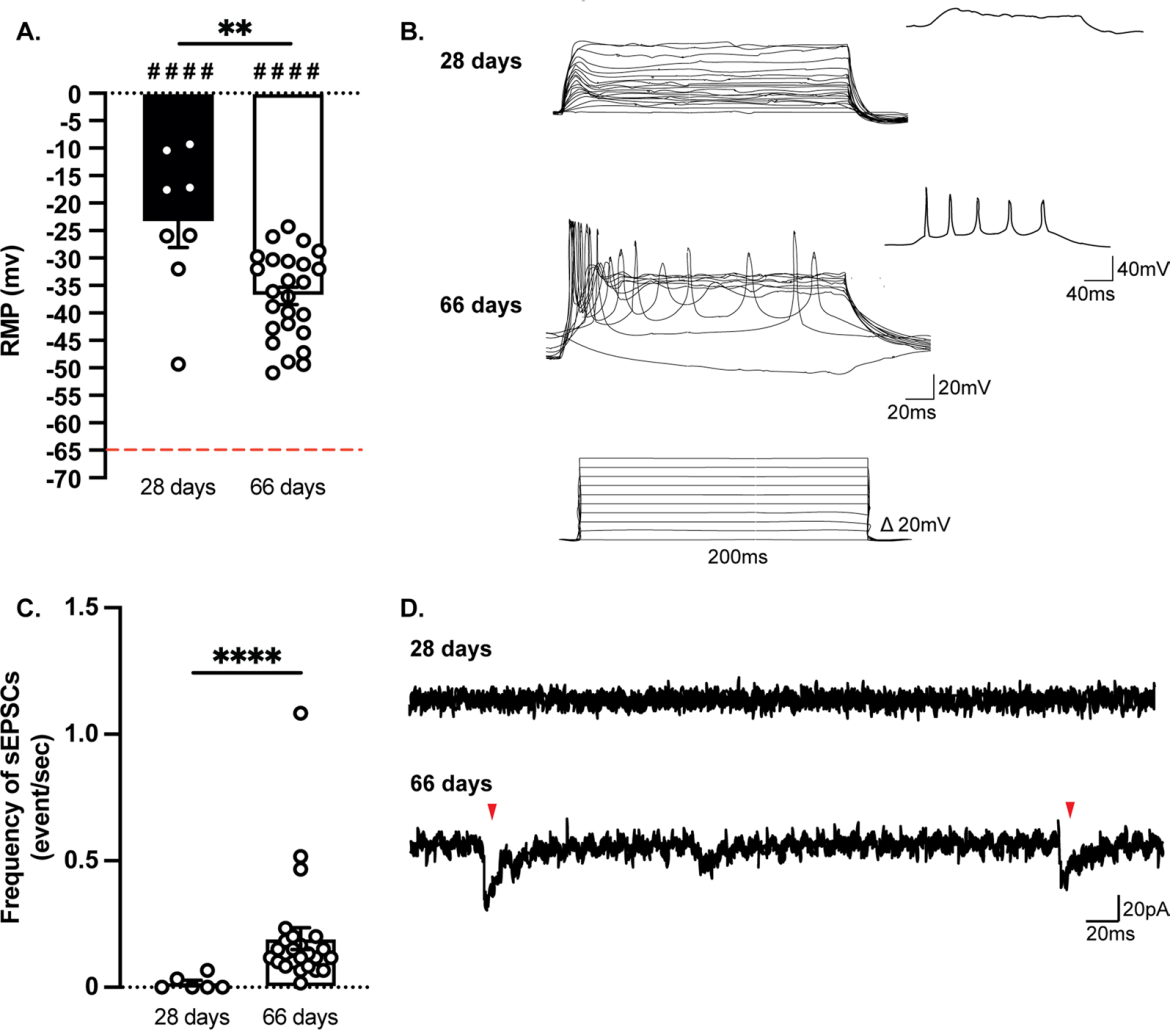
Fully active differentiated neurons can be obtained after 66 days of monoculture. **A** The resting membrane potential was significantly higher than the theoretical value (− 65 mV, red dotted line) both at 28 and 66 days. The RMP at 66 days was significantly lower compared to 28 days. **B** Representative traces of firing events after injecting increasing current in the patching pipette. **C** The frequency of sEPSCs was significantly higher at 66 days. **D** Representative traces of gap-free recordings. The red arrowheads indicate the position of EPSCs (Results are shown as mean ± S.E.M.; 28 days: *n* = 8 cells out of one batch, 66 days: *n* = 18 cells out of two batches; ^####^*p* < 0.0001: significantly different from − 65 mV; **p < 0.01, ****p < 0.0001: significantly different from the 28-day condition)

The first step in characterizing our hiPSC-derived neurons was to verify their functional maturity. Fully mature hiPSC-derived neurons can be obtained after 28 days of culture, as previously reported [30]. However, in our 28-day cultures, a significantly higher resting membrane potential (RMP) was observed compared to the theoretical value of − 65 mV (Fig. 5A). Also, no action potentials were detected at stepwise current injections (Fig. 5B) and only a very low frequency of sEPSCs could be recorded (Fig. 5C, D). When cultures were allowed to mature for additional 38 days (66 days in total), the resting membrane potential was significantly lower compared to the 28-day neurons and current injections triggered the firing of action potentials (Fig. 5A). Moreover, intracellular current-clamp recordings showed an increased frequency of sEPSCs, compared to 28-day neurons (Fig. 5C). One hypothesis is that sodium channels, which are necessary for the generation of action potentials, are expressed late in the maturation process (Additional file 1: Fig. S2). Therefore, we used fully mature neuronal cultures that were differentiated for 66 days for all the remaining experiments.

### Control astrocytes increase neuronal synaptic activity

**Fig. 6:**
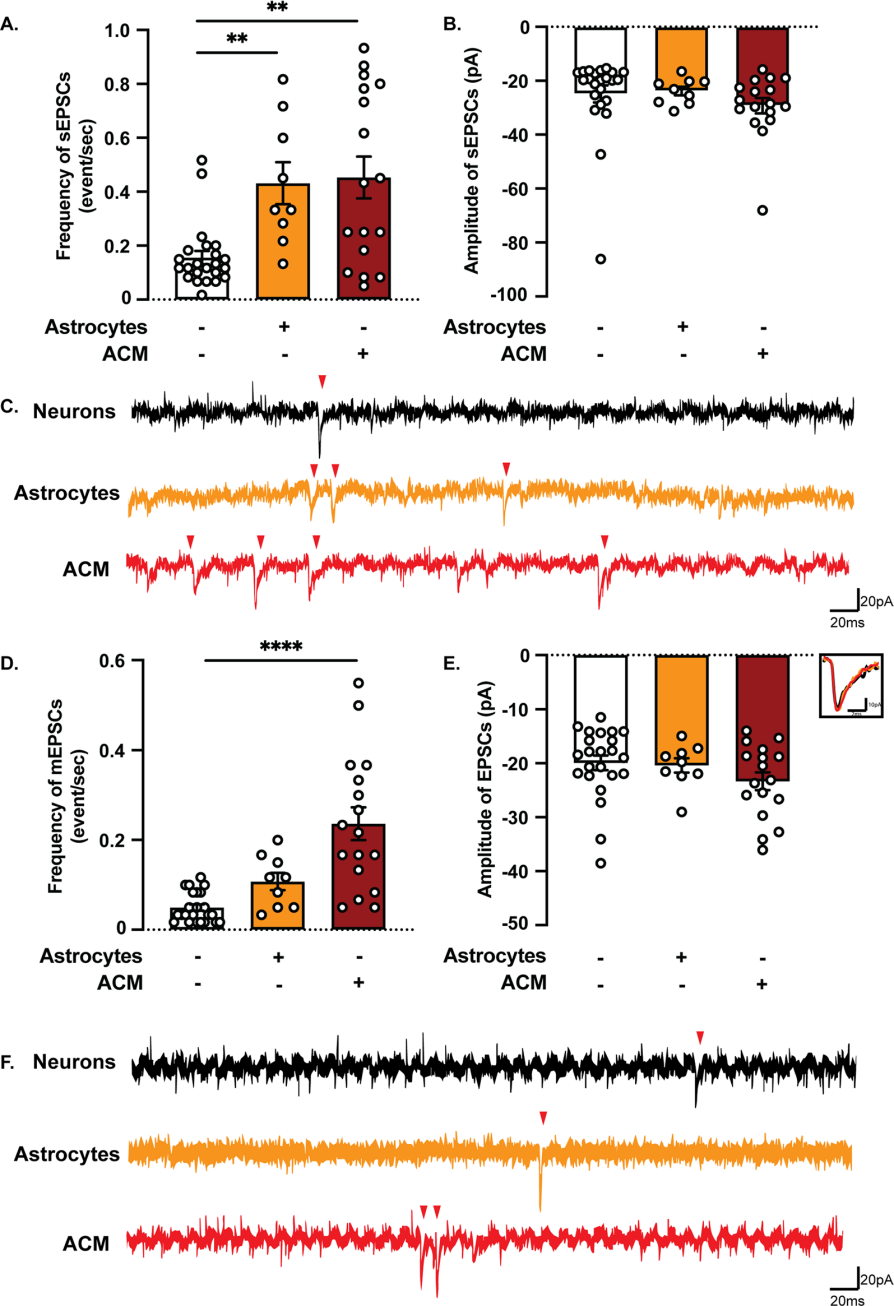
Astrocytes affect the frequency, but not the amplitude, of both sEPSCs and mEPSCs*.* **A** Both astrocytes and ACM increased the frequency of sEPSCs compared to control neuronal monocultures. **B** The amplitude of sEPSCs was not affected by the presence of astrocytes or ACM. **D** Under TTx 0.5 µM treatment, only the ACM increased the frequency of mEPSCs compared to neuronal monocultures. **E** The amplitude of mEPSCs was not affected by the presence of astrocytes or ACM. **C**, **F** Representative traces of gap-free recordings. Red arrowheads indicate the position of EPSCs. (Results are shown as mean ± S.E.M.; neurons: *n* = 23 cells out of two batches, neurons + astrocytes: *n* = 9 cells out of one batch, neurons + ACM: *n* = 17 cells out of two batches; **p < 0.01, ****p < 0.0001: significantly different from neuronal monocultures)

We then sought to investigate whether the direct contact with astrocytes or the addition of conditioned media from astrocytic cultures would affect neuronal activity. We recorded synaptic activity in neuronal monocultures, co-cultures with control astrocytes and neuronal monocultures treated with ACM from control astrocytes. There was no statistical difference in the resting membrane potential between the different cultures, although all conditions were slightly depolarized compared to the theoretical value of -65 mV (Additional file 1: Fig. S3A). Notably, analysis of sEPSCs revealed an increased frequency, but not amplitude, in co-cultures and neurons treated with control ACM, compared to neurons only (Fig. 6A–C). To further assess the effect on synaptic transmission, mEPSCs were recorded in the presence of TTx that blocks action potentials and allows for assessment of the properties of individual synapses without any network activity. As expected, neurons cultured in control ACM showed an increased frequency, but not amplitude of mEPSCs, compared to control neurons (Fig. 6D–F). However, neurons in co-cultures were not significantly different from neuronal monocultures. Altogether, our observations support the idea that gliotransmitters secreted into the ACM facilitate synaptic release, leading to an increased frequency of synaptic events.

### Direct contact of Aβ astrocytes with neurons alters neuronal synaptic activity

**Fig. 7:**
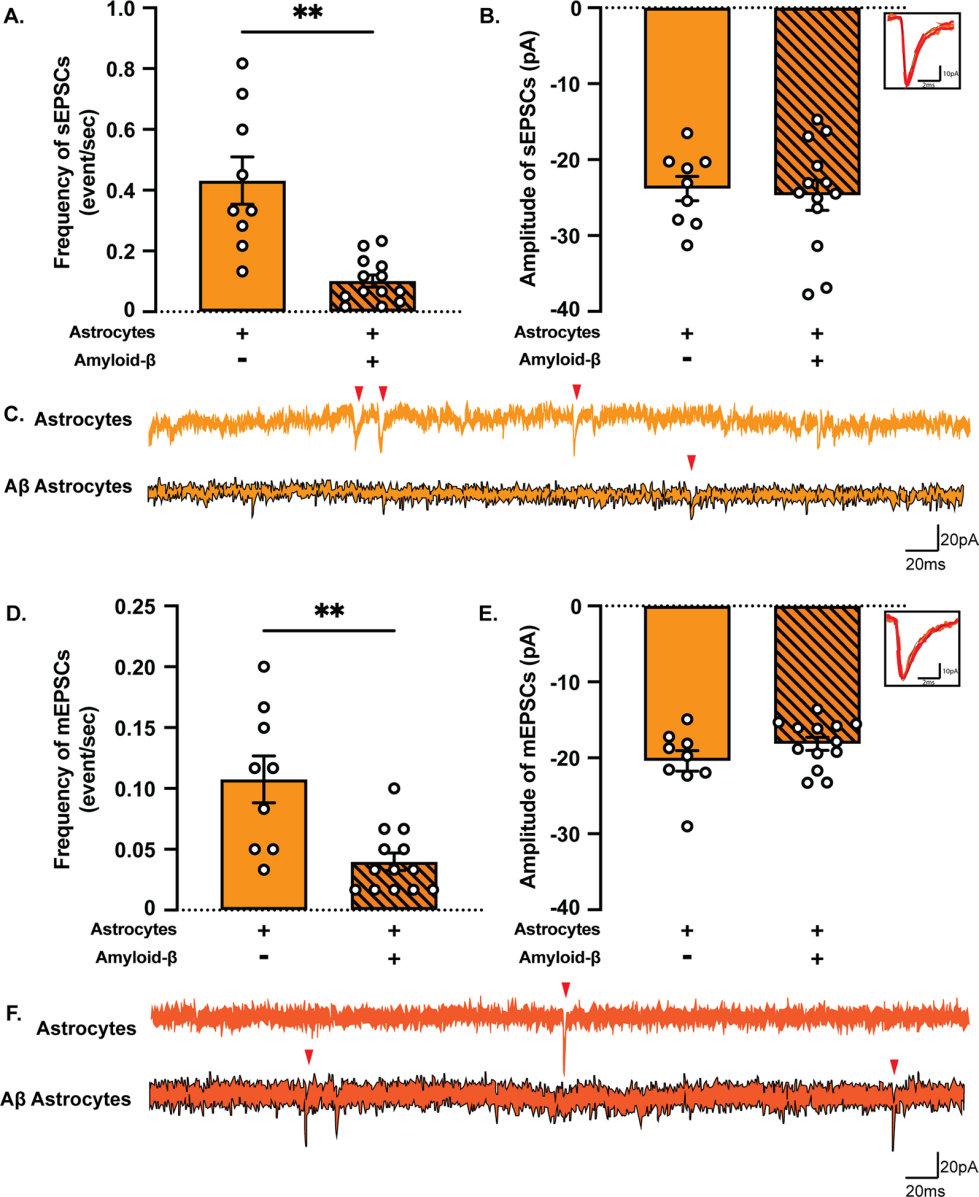
Co-culturing with Aβ-exposed astrocytes decreases neuronal synaptic activity. **A** The frequency of sEPSCs was significantly decreased in Aβ co-cultures. **B** No difference was detected in the amplitude of sEPSCs. **D** A similar decrease was also observed in the Aβ co-culture frequency of mEPSCs. **E** There was no difference in the amplitude of mEPSCs in control co-cultures compared to Aβ co-cultures. **C**, **F** Representative traces of gap-free recordings. Red arrowheads indicate the position of sEPSCs and mEPSCs, respectively. (Results are presented as mean ± S.E.M.; neurons + astrocytes: *n* = 9 cells out of one batch, neurons + Aβ astrocytes: *n* = 13 cells out of one batch; **p < 0.01)

Aggregation of Aβ is believed to be a major cause of neuronal impairment in AD and is known to affect neuronal function long before inducing cell death [35]. However, the way through which Aβ pathology interferes with the astrocytic capacity to support synaptic function is still unclear. To that end, we investigated neuronal activity in co-cultures of neurons and astrocytes with or without Aβ inclusions (Fig. 7). Again, there was no significant difference in the resting membrane potential between control and Aβ co-cultures (Additional file 1: Fig. S3B). The frequency of sEPSCs (Fig. 7A–C) and mEPSCs (Fig. 7D–F) was decreased in Aβ co-cultures compared to control co-cultures without any difference in amplitude.

### Conditioned medium from Aβ-exposed astrocytes increases neuronal synaptic activity

**Fig. 8:**
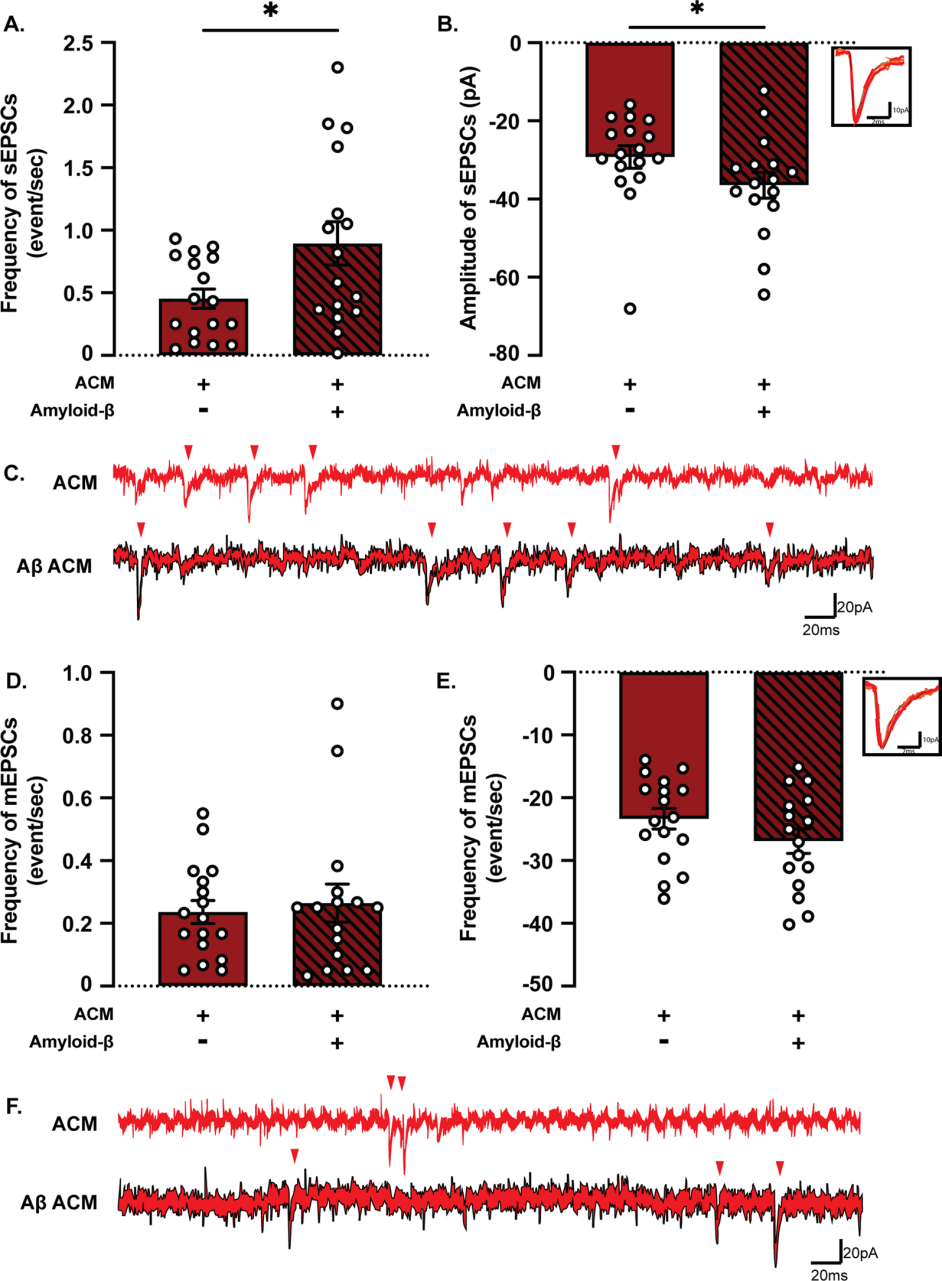
Conditioned media from astrocytes with Aβ inclusions increase neuronal synaptic activity*.* **A** The frequency of sEPSCs was significantly increased in neuronal monocultures treated with Aβ ACM compared to monocultures treated with control ACM. **B** The amplitude of sEPSCs was also increased in Aβ ACM cultures. **D**, **E** No difference was observed in the frequency and amplitude of mEPSCs in monocultures exposed to Aβ ACM compared to cultures exposed to control ACM. **C**, **F** Representative traces of gap-free recordings. Red arrowheads indicate the position of sEPSCs and mEPSCs, respectively. (Results are presented as mean ± S.E.M.; neurons + ACM: *n* = 17 cells out of two batches, neurons + Aβ ACM: *n* = 16 cells out of two batches; *p < 0.05)

The reduced frequency of synaptic events in the presence of Aβ-exposed astrocytes could result from the effect of Aβ inclusions on the release of gliotransmitters that, under physiological conditions, support synaptic transmission. To test this hypothesis, we assessed the activity in neuronal monocultures treated with ACM from control or Aβ-exposed astrocytes. There was again no difference in RMP between neurons treated with control or Aβ ACM (Additional file 1: Fig. S3B). In contrast to the co-culture data, both the frequency and amplitude of sEPSCs was increased in neurons with Aβ ACM compared to neurons with control ACM (Figs. 8A–C). No differences were observed in neither mEPSCs frequency nor amplitude between neurons treated with control or Aβ ACM (Fig. 8D–F), suggesting that the presence of astrocytes affects network activity by more mechanisms than just the secretion of gliotransmitters. This was further confirmed by a statistically significant increase in both sEPSC and mEPSC frequency and amplitude in neurons treated with Aβ ACM compared to Aβ co-cultures (Additional file 1: Fig. S4).

## Discussion

Astrocytes are crucial for maintaining brain homeostasis, but they are also highly involved in neuroinflammation. In the pathological brain, astrocytes, similar to microglia, enter a reactive, inflammatory state that has the potential to harm nearby cells, especially neurons [36]. Accumulating evidence indicates that astrocytes can play a central role during AD progression, by promoting cell-to-cell spreading of pathogenic protein aggregates and secretion of proinflammatory mediators [37–39]. In addition, reactive astrocytes may exacerbate AD pathology, by neglecting their main role in supporting neurons. Indeed, gene and protein expression studies have revealed differences in key molecules between reactive and naïve astrocytes that suggest a likely shift in function [40–42].

We have previously demonstrated that astrocytes can engulf large amounts of aggregated Aβ, but then accumulate, rather than degrade the ingested material. The accumulation of Aβ in murine astrocytes causes lysosomal dysfunction, formation of extremely enlarged endosomes, release of EVs containing neurotoxic Aβ species and increased levels of ApoE [20, 43]. More specifically, our data demonstrated that EVs isolated from astrocytes treated with soluble Aβ aggregates induced synaptic loss, axonal swelling and vacuolization of the neuronal cell bodies, which consequently led to apoptosis of primary murine cortical neurons [25].

Although studies in animal models as well as cultured mouse cells have been crucial for the understanding of AD, it is important to remember that the mouse and human brains differ in many ways, both at the macroscopic and cellular level. This is especially clear when it comes to astrocytes that show distinct morphology, size and complexity [44, 45]. In the present study, we aimed to bridge the gap between human pathology and in vitro systems by modeling AD processes, using previously established protocols to generate hiPSC-derived neurons and astrocytes [30, 31].

To date, numerous protocols have been developed for the differentiation of hiPSCs into astrocytes and neurons that differ in timing and media formulations (reviewed in [46] and [47]). A common point of concern has been the maturity of the generated cells. Our hiPSC-derived neurons and astrocytes expressed all lineage-specific, mature markers, indicating successful differentiation from the neuronal progenitor state. Our next step was to assess the human astrocytes’ ability to engulf Aβ-F and evaluate the effect of Aβ inclusions in cell functionality and potential role in AD spreading.

After confirming that the astrocytes take up Aβ-F, we developed a co-culture system of neurons and astrocytes to study their interactions. The introduction of Aβ-containing astrocytes into the neuronal cultures led to increased clearance of clumps of apoptotic cells. Previous studies have shown that the presence of Aβ shifts the astrocytes towards a more reactive state that could explain the increased phagocytic capacity in our culture model [48]. Moreover, reactive astrocytes have been demonstrated to engulf dead cells, damaged synapses and protein aggregates more effectively compared to astrocytes from the healthy brain [18, 49, 50].

Although acute inflammation is crucial for removal of dead cells and pathogens, a constant activation of the immune system is detrimental to the tissue. Increasing evidence suggests that pathological processes in AD are largely due to chronic inflammation [38, 51]. Aggregated Aβ and tau trigger an innate immune response, resulting in astrocytic and microglial release of harmful inflammatory mediators. Conditioned media from control, but not Aβ-exposed astrocytes, provided support to neuronal monocultures, which translated to increased viability compared to control neuronal monocultures. However, in contrast to our previous data from murine cultures [20], EVs isolated from human, Aβ-exposed astrocytes, did not show any increased neurotoxicity, compared to control EVs. This may be explained by a more toxic content in the mouse EVs or a more effective EV uptake in murine neurons compared to human neurons. It is also possible that some trophic factors are present in the human EVs that compensate for the toxic effect.

Astrocytes secrete a wide array of classic neuroactive molecules and hormones, as well as metabolic, trophic and plastic factors [37]. In addition, they release lipids and lipid binding proteins and neurons completely rely on astrocytes for their cholesterol supply. Hence, maintaining astrocytic functionality might be crucial in limiting disease propagation.

In particular, astrocytic function is important for keeping synaptic transmission balanced and previous reports suggest that astrocytes are essential for the functional maturation of hiPSC-derived neurons in vitro [30, 52]. In our study, hiPSC-derived neurons displayed normal activity patterns after 66 days of culture without the presence of astrocytes. Patch-clamp analysis revealed that both astrocytes and ACM enhance the frequency of sEPSCs, emphasizing the important role of astrocytes in network maturation [8, 53]. These data support previous findings that astrocytes can modify synaptic activity through both direct and indirect contact with neurons [54, 55].

Our results demonstrate that ACM increases the frequency of both sEPSCs and mEPSCs, strongly supporting the gliotransmission hypothesis [13]. Astrocytes can release many factors that are known to enhance synaptic transmission, including glutamate, D-serine, ATP and various growth factors [56–58]. Interestingly, ACM was more effective than co-culturing when it came to increasing mEPSC frequency. This finding could be explained by the synaptic pruning that may take place in the co-cultures, leading to fewer active synapses [13, 56]. Further investigations are necessary to decipher which gliotransmitters are expressed in the hiPSC-derived cultures.

Next, we sought to examine how Aβ accumulation in human astrocytes affects synaptic activity in our cell culture model of AD. Notably, sEPSC frequency was significantly reduced in co-cultures where astrocytes had been exposed to Aβ, compared to co-cultures with control astrocytes. We hypothesize that astrocytes, while displacing their activity to Aβ clearance, reduce their support to synaptic activity [59]. However, only sEPSCs, that are triggered by action potentials, are affected, and not mEPSCs. Since mEPSCs are a measurement of stochastic presynaptic release events that do not require triggering by action potentials, they reflect the activity level at individual synapses, independently of the network or intrinsic properties of the cell. Thus, it is unlikely that reduced support of synapse formation and maturation is the only explanation for the effect of Aβ.

Hippocampal hyperactivity has been observed at the early stages of AD [60–62], whereas synaptic loss and reduced activity is prominent at a later state of the disease [63]. Astrocytes with Aβ inclusions seem to release factors that affect intrinsic properties of neurons, making the network more active. This hypothesis is supported by the fact that Aβ ACM increases the sEPSC frequency even further, compared to the actual presence of Aβ-exposed astrocytes.

## Conclusions

Taken together, our results demonstrate that intracellular deposits of aggregated Aβ affect the reactivity state of astrocytes, as well as their ability to support neuronal function via both direct interaction and releasable factors. We believe that data from this study will contribute to deciphering the neuron–astrocyte crosstalk in AD phenotype propagation.

## Supplementary Information

**Additional file 1: Figure S1.** Representative MAP2 expression in neurons from different experimental conditions Representative ICC images from MAP2+ neurons in control monocultures, control co-cultures and Aβ co-cultures, respectively (scale bar: 200 μm). **Figure S2. **Neurons from 28-day old cultures lack inward sodium current*. *Increasing voltages were applied throughout the patching pipette and intracellular current was measured. Once the action potential threshold was reached (around -40 mV), the intracellular current of 66-day old cells (clear circles) rapidly increased, characteristic of an inward sodium current. This increase was not observed in 28-day old cells (black circles) (Results are presented as mean ± S.E.M.; 28 days: *n*=8 cells out of one batch, 66 days: *n*=18 cells out of two batches). **Figure S3.** Direct or indirect presence of astrocytes does not affect the resting membrane potential of neurons*. *A Regardless of the presence of astrocytes or ACM, the RMP was unchanged between the groups. The RMP was still significantly higher than the theoretical value of -65 mV. B When astrocytes were exposed to Aβ, the resting RMP was still unchanged between groups and higher than -65 mV (Results are presented as mean ± S.E.M.; neurons: *n*=23 cells out of two batches; neurons + astrocytes: *n*=9 cells out of one batch, neurons + Aβ astrocytes: *n*=13 cells out of one batch, neurons + ACM: *n*=17 cells out of two batches, neurons + Aβ ACM: *n*=16 out of two batches; # # # # *p*<0.0001: significantly different from -65 mV). **Figure S4.** Differential effect of Aβ in neuronal activity via physical or remote presence*. *A The frequency of sEPSCs was significantly increased in neuronal monocultures treated with Aβ ACM compared to Aβ co-cultures. B The amplitude of sEPSCs was also increased in Aβ ACM treated cultures compared to Aβ co-cultures. C, D Similar increase was also observed in the frequency and amplitude of mEPSCs (Results are presented as mean ± S.E.M.; neurons + Aβ astrocytes: *n*=13 cells, neurons + Aβ ACM: *n*=16 cells; **p<0.01, ***p<0.001, ****p<0.0001). **Table S1.** Statistical tests used in this study. **Table S2.** Variation between the groups does not affect the statistical analyses

## Data Availability

Not applicable.

## Abbreviations

ACM: Astrocyte conditioned medium
AD: Alzheimer’s disease
Aβ/Aβ-F: Amyloid-beta/amyloid-beta fibrils
bFGF: Basic fibroblast growth factor
CNTF: Ciliary neurotrophic factor
DIV: Days in vitro
EGTA: Ethylene glycol tetra acetic acid
EPSCs (m/s): Excitatory post-synaptic currents (mini/spontaneous)
EVs: Extracellular vesicles
hiPSCs: Human induced pluripotent stem cells
IGF-1: Insulin growth factor-1
ltNES cells: Long-term neuroepithelial-like stem cells
NGS: Normal goat serum
PBS: Phosphate-buffered saline
PFA: Paraformaldehyde
RMP: Resting membrane potential
RT: Room temperature
TEM: Transmission electron microscopy
TNTs: Tunneling nanotubes
TTx: Tetrodotoxin
