# Supplementary material for: Intracellular deposits of amyloid-beta influence the ability of human iPSC-derived astrocytes to support neuronal function
Source: J Neuroinflammation. 2023 Jan 3;20:3. doi: 10.1186/s12974-022-02687-5 (PMC9809017; doi:10.1186/s12974-022-02687-5)
Supplement: Supplementary file 1 — Additional file 1: Figure S1. Representative MAP2 expression in neurons from different experimental conditions Representative ICC images from MAP2+ neurons in control monocultures, control co-cultures and Aβ co-cultures, respectively (scale bar: 200 μm). Figure S2. Neurons from 28-day old cultures lack inward sodium current. Increasing voltages were applied throughout the patching pipette and intracellular current was measured. Once the action potential threshold was reached (around -40 mV), the intracellular current of 66-day old cells (clear circles) rapidly increased, characteristic of an inward sodium current. This increase was not observed in 28-day old cells (black circles) (Results are presented as mean ± S.E.M.; 28 days: n=8 cells out of one batch, 66 days: n=18 cells out of two batches). Figure S3. Direct or indirect presence of astrocytes does not affect the resting membrane potential of neurons. A Regardless of the presence of astrocytes or ACM, the RMP was unchanged between the groups. The RMP was still significantly higher than the theoretical value of -65 mV. B When astrocytes were exposed to Aβ, the resting RMP was still unchanged between groups and higher than -65 mV (Results are presented as mean ± S.E.M.; neurons: n=23 cells out of two batches; neurons + astrocytes: n=9 cells out of one batch, neurons + Aβ astrocytes: n=13 cells out of one batch, neurons + ACM: n=17 cells out of two batches, neurons + Aβ ACM: n=16 out of two batches; # # # # p<0.0001: significantly different from -65 mV). Figure S4. Differential effect of Aβ in neuronal activity via physical or remote presence. A The frequency of sEPSCs was significantly increased in neuronal monocultures treated with Aβ ACM compared to Aβ co-cultures. B The amplitude of sEPSCs was also increased in Aβ ACM treated cultures compared to Aβ co-cultures. C, D Similar increase was also observed in the frequency and amplitude of mEPSCs (Results are presented as mean ± S.E.M.; neurons + Aβ astrocytes: n=1 [file 12974_2022_2687_MOESM1_ESM.docx]

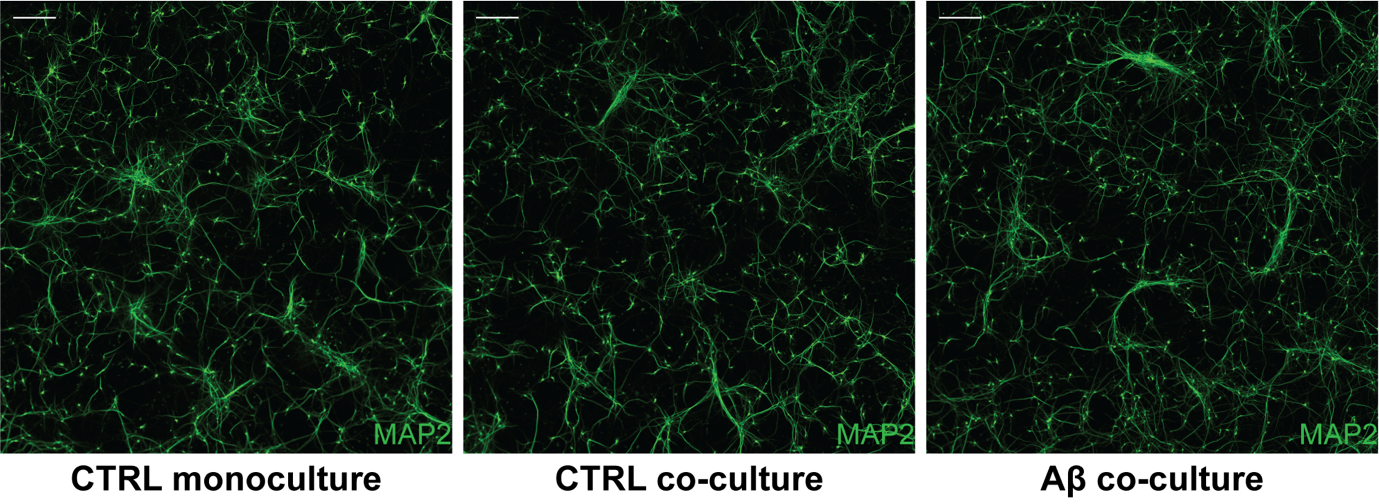


**Figure S1.** Expression of the neuronal marker MAP2

Representative MAP2 expression in control monocultures, control co-cultures and Aβ co-cultures, respectively (scale bar: 200 μm).


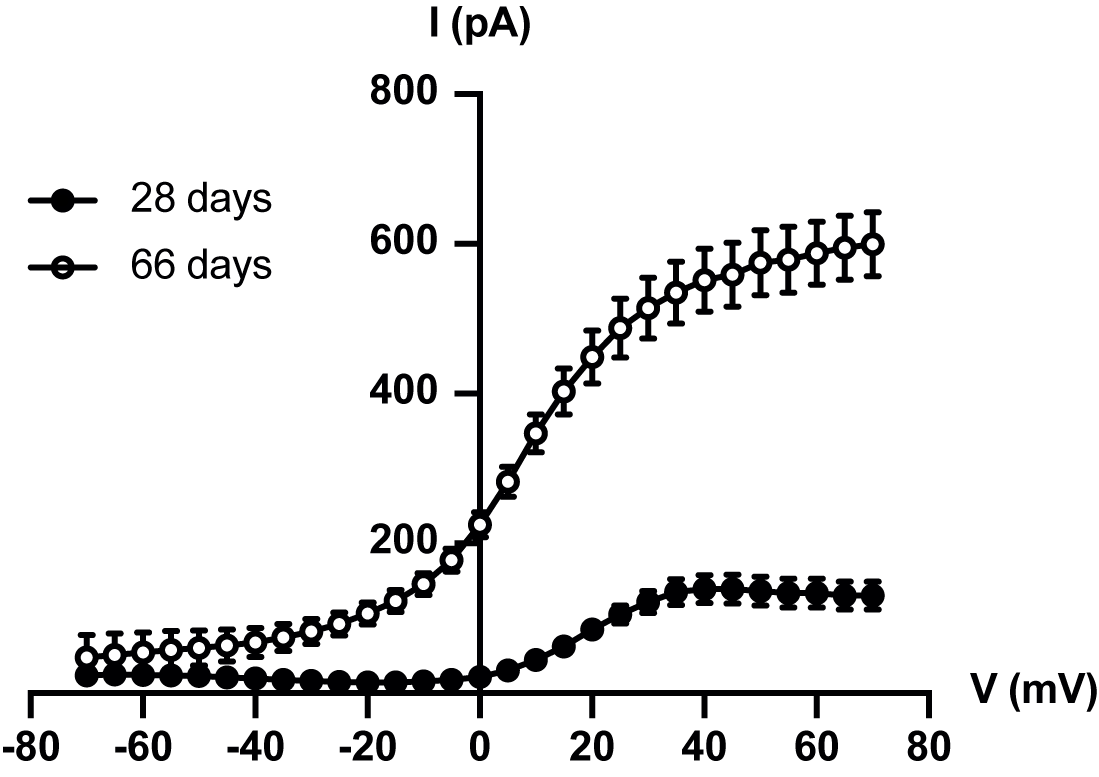


**Figure S2.** Neurons from 28-day old cultures lack inward sodium current

Increasing voltages were applied throughout the patching pipette and intracellular current was measured. Once the action potential threshold was reached (around -40 mV), the intracellular current of 66-day old cells (clear circles) rapidly increased, characteristic of an inward sodium current. This increase was not observed in 28-day old cells (black circles) (Results are presented as mean ± S.E.M.; 28 days: n=8 cells out of one batch, 66 days: n=18 cells out of two batches).


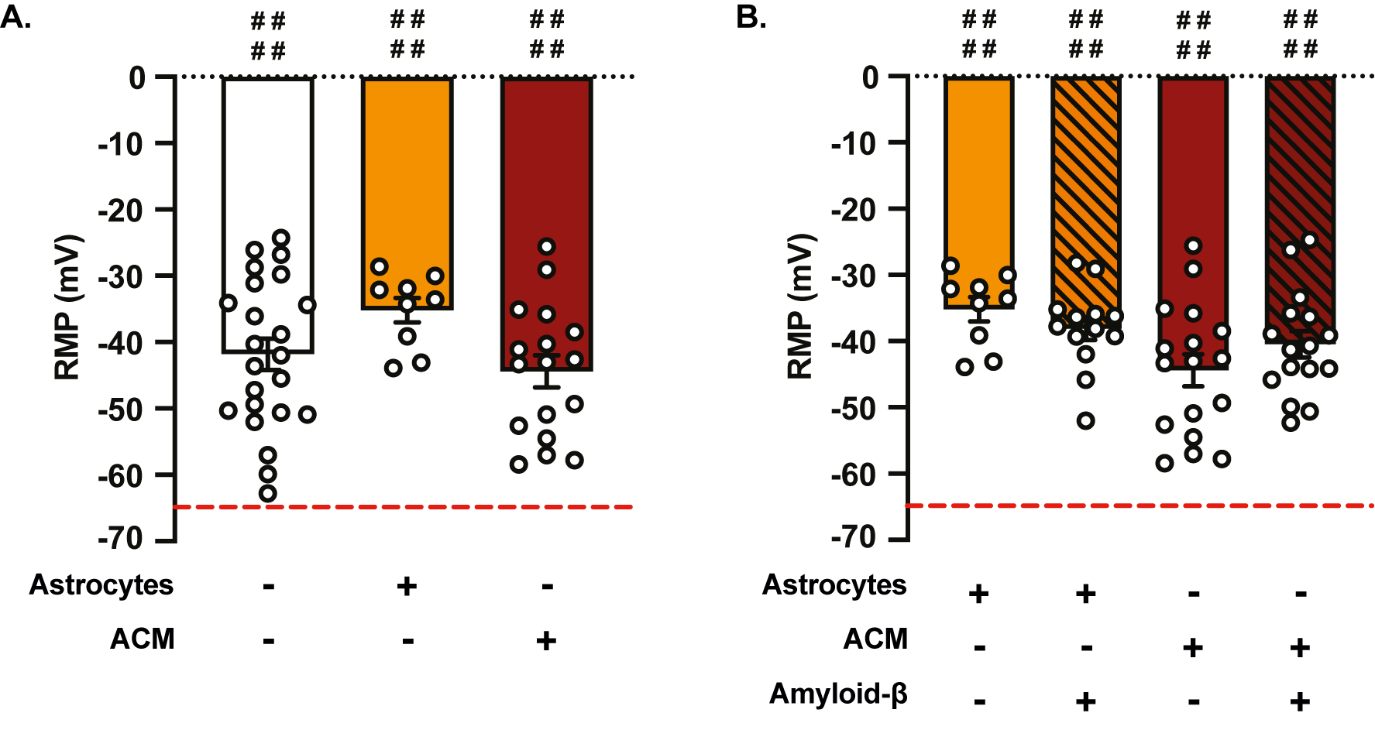


**Figure S3.** Direct or indirect presence of astrocytes does not affect the resting membrane potential of neurons

**A** Regardless of the presence of astrocytes or ACM, the RMP was unchanged between the groups. Thee RMP was still significantly higher than the theoretical value of -65 mV. **B** When astrocytes were exposed to Aβ, the resting RMP was still unchanged between groups and higher than -65 mV (Results are presented as mean ± S.E.M.; neurons: n=23 cells out of two batches; neurons + astrocytes: n=9 cells out of one batch, neurons + Aβ astrocytes: n=13 cells out of one batch, neurons + ACM: n=17 cells out of two batches, neurons + Aβ ACM: n=16 out of two batches; ####p<0.0001: significantly different from -65 mV).


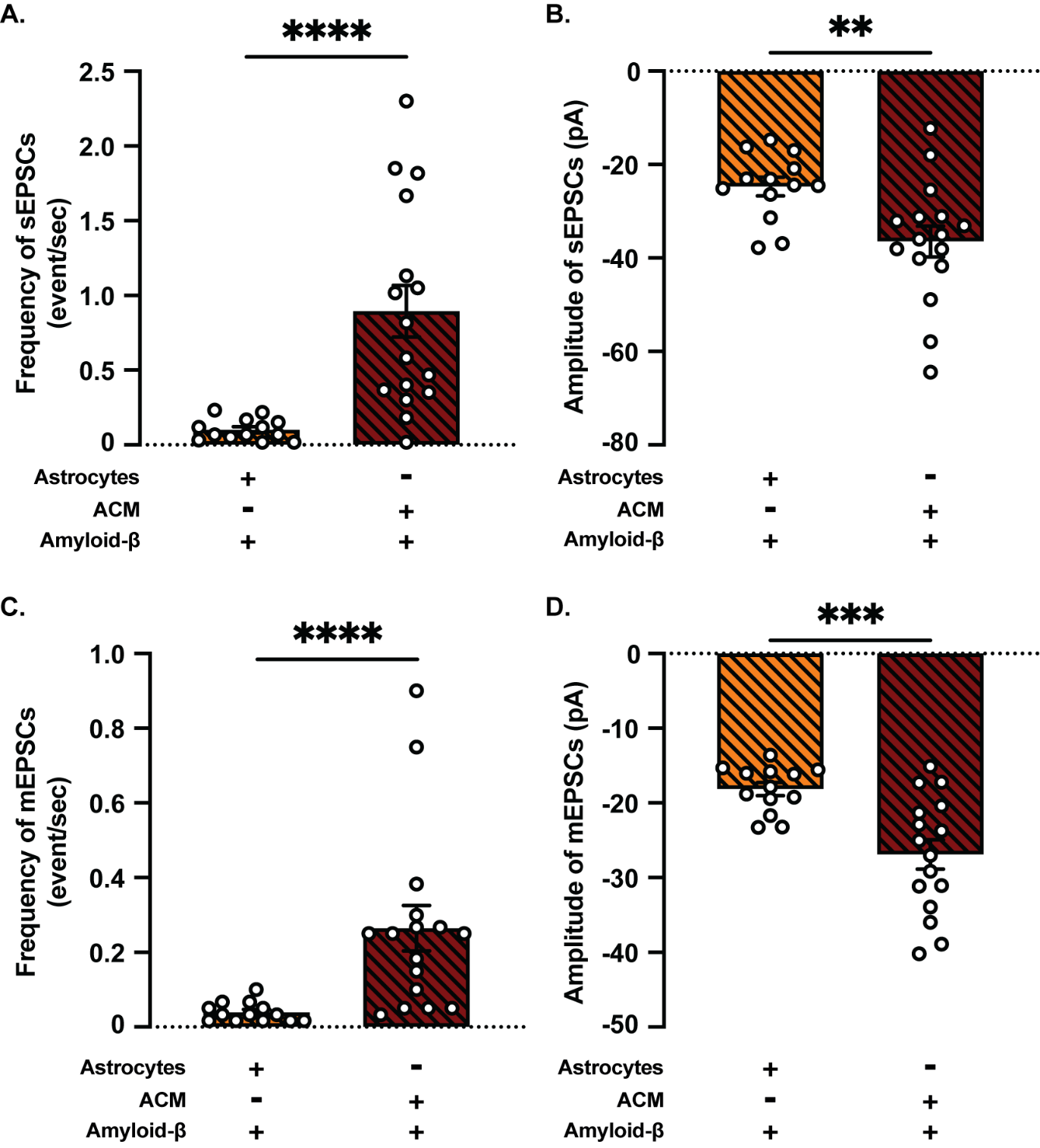


**Figure S4.** Differential effect of Aβ in neuronal activity via physical or remote presence

**A** The frequency of sEPSCs was significantly increased in neuronal monocultures treated with Aβ ACM compared to Aβ co-cultures. **B** The amplitude of sEPSCs was also increased in Aβ ACM treated cultures compared to Aβ co-cultures. **C, D** Similar increase was also observed in the frequency and amplitude of mEPSCs (Results are presented as mean ± S.E.M.; neurons + Aβ astrocytes: n=13 cells, neurons + Aβ ACM: n=16 cells; **p<0.01, ***p<0.001, ****p<0.0001).

| **Table S1.** Statistical tests used in this study | | | |
| --- | --- | --- | --- |
|  | **Test** | **Value** | ***p*-value** |
| **Figure 3** |  |  |  |
| Viability of cultures | Brown-Forsythe and Welch one-way ANOVA | 14.33 and 18.17 | <0.0001 |
| Vimentin+ area% | Unpaired t-test | 1.653 | 0.1049 |
| MAP2+ area% | One-way ANOVA | 3.397 | 0.0389 |
| **Figure 4** |  |  |  |
| (C) Viability of cultures | Kruskal-Wallis test | 23.54 | <0.0001 |
| (D) Viability of cultures | Unpaired t-test | 1.280 | 0.2081 |
| **Figure 5** |  |  |  |
| Resting membrane potential (compared to -65 mV) | One-sample t-test | 28 days: 8.982 | 28 days: <0.001 |
|  |  | 66 days: 17.884 | 66 days: <0.001 |
| Resting membrane potential | Unpaired t-test | 3.560 | <0.001 |
| Frequency of sEPSCs | Mann-Whitney test | 5 | <0.001 |
| **Figure 6** |  |  |  |
| Frequency of sEPSCs | Kruskal-Wallis test | 14.034 | <0.001 |
| Amplitude of sEPSCs | One-way ANOVA | 0.757 | 0.475 |
| Frequency of mEPSCs | Kruskal-Wallis test | 24.967 | <0.001 |
| Amplitude of mEPSCs | Kruskal-Wallis test | 3.303 | 0.192 |
| **Figure 7** |  |  |  |
| Frequency of sEPSCs | Unpaired t-test with Welch's correction | 4.114 | 0.0026 |
| Amplitude of sEPSCs | Unpaired t-test | 0.3206 | 0.7518 |
| Frequency of mEPSCs | Unpaired t-test with Welch's correction | 3.290 | 0.0079 |
| Amplitude of mEPSCs | Unpaired t-test | 1.471 | 0.1569 |
| **Figure 8** |  |  |  |
| Frequency of sEPSCs | Unpaired t-test with Welch's correction | 2.333 | 0.0298 |
| Amplitude of sEPSCs | Mann-Whitney test | 75 | 0.0279 |
| Frequency of mEPSCs | Mann-Whitney test | 132 | 0.08938 |
| Amplitude of mEPSCs | Unpaired t-test | 1.393 | 0.1735 |
| **Figure S3** |  |  |  |
| (A) Resting membrane potential (compared to -65 mV) | One-sample t-test | Neuronss:9.848 | Neurons:<0.001 |
|  |  | Neurons+Astrocytes:16.076 | Neurons+Astrocytes:<0.001 |
|  |  | Neurons+ACM:8.527 | Neurons+ACM:<0.001 |
| (A) Resting membrane potential | One-way ANOVA | 2.506 | 0.093 |
| (B) Resting membrane potential (compared to -65 mV) | One-sample t-test | Neurons+Astrocytes:16.076 | Neurons+Astrocytes:<0.001 |
|  |  | Neurons+Aβ Astrocytes:15.515 | Neurons+Aβ Astrocytes<0.001 |
|  |  | Neurons+ACM:8.527 | Neurons+ACM:<0.001 |
|  |  | Neurons+Aβ ACM:12.370 | Neurons+Aβ ACM:<0.001 |
| (B) Resting membrane potential | One-way ANOVA | Neurons+Astrocytes:16.076 | Neurons+Astrocytes:16.076 |
|  |  | Neurons+Aβ Astrocytes:15.515 | Neuron+Aβ Astrocytes:15.515 |
|  |  | Neurons+ACM:8.527 | Neurons+ACM:8.527 |
|  |  | Neurons+Aβ ACM:12.370 | Neurons+Aβ ACM:12.370 |
| **Figure S4** |  |  |  |
| Frequency of sEPSCs | Mann-Whitney test | 15 | <0.0001 |
| Amplitude of sEPSCs | Welch's t test | 3.066 | 0.0053 |
| Frequency of mEPSCs | Mann-Whitney test | 19 | <0.0001 |
| Amplitude of mEPSCs | Welch's t test | 4.080 | 0.0006 |
| **Table S2** |  |  |  |
| Frequency of sEPSCs | Asymptotic test for equality of CV | 0.630 | 0.959 |
| Amplitude of sEPSCs | Asymptotic test for equality of CV | 12.44 | 0.014 |
| Frequency of mEPSCs | Asymptotic test for equality of CV | 2.158 | 0.706 |
| Amplitude of mEPSCs | Asymptotic test for equality of CV | 5.748 | 0.218 |

**Table S2.** Variation between the groups does not affect the statistical analyses

|  | **Neurons + astrocytes** | **Neurons +  Aβ astrocytes** | **Neurons + ACM** | **Neurons +  Aβ ACM** | **Asymptotic test** |
| --- | --- | --- | --- | --- | --- |
| Frequency of sEPSC | 54.03% | 71.54% | 70.65% | 77.25% | p=0.959 |
| Amplitude of sEPSC | 20.06% | 29.09% | 40.70% | 35.98% | p=0.014 |
| Frequency of mEPSCs | 58.83% | 65.28% | 63.79% | 92.26% | p=0.706 |
| Amplitude of mEPSC | 19.78% | 17.25% | 29.14% | 29.13% | p=0.218 |

Coefficient of variation (CV=$\frac{\sigma}{\mu}\times100$) was calculated for every group for both frequency and amplitude of sEPSCs and mEPSCs. An asymptotic test for comparison of coefficient of variation of k populations revealed a significant difference in CVs calculated for the amplitude of sEPSCs. Overall, the variation did not affect the statistical analyses (ACM = astrocyte conditioned media).
